# Assessing the ecological validity of numerosity-selective neuronal populations with real-world natural scenes

**DOI:** 10.1016/j.isci.2022.105267

**Published:** 2022-10-04

**Authors:** Shir Hofstetter, Serge O. Dumoulin

**Affiliations:** 1 The Spinoza Centre for Neuroimaging, Amsterdam, the Netherlands; 2 Department of Computational Cognitive Neuroscience and Neuroimaging, Netherlands Institute for Neuroscience, Amsterdam, the Netherlands; 3 Department of Experimental Psychology, Helmholtz Institute, Utrecht University, Utrecht, the Netherlands; 4 Department of Experimental and Applied Psychology, VU University, Amsterdam, the Netherlands

**Keywords:** Biological sciences, Neuroscience, Cognitive neuroscience

## Abstract

Animals and humans are able to quickly and effortlessly estimate the number of items in a set: their numerosity. Numerosity perception is thought to be critical to behavior, from feeding to escaping predators to human mathematical cognition. Virtually, all scientific studies on numerosity mechanisms use well controlled but artificial stimuli to isolate the numerosity dimension from other physical quantities. Here, we probed the ecological validity of these artificial stimuli and evaluate whether an important component in numerosity processing, the numerosity-selective neural populations, also respond to numerosity of items in real-world natural scenes. Using 7T MRI and natural images from a wide range of categories, we provide evidence that the numerosity-tuned neuronal populations show numerosity-selective responses when viewing images from a real-world natural scene. Our findings strengthen the role of numerosity-selective neurons in numerosity perception and provide an important link to their function in numerosity perception in real-world settings.

## Highlights

• Numerosity is typically studied using well-controlled visual stimuli. i.e. dots
• Using dot stimuli, neural populations respond selectively to preferred numerosities
• Numerosity-selective neural populations also respond to natural images
• These neural populations respond to the numerosity of items in natural images

## Graphical abstract

**Figure:**
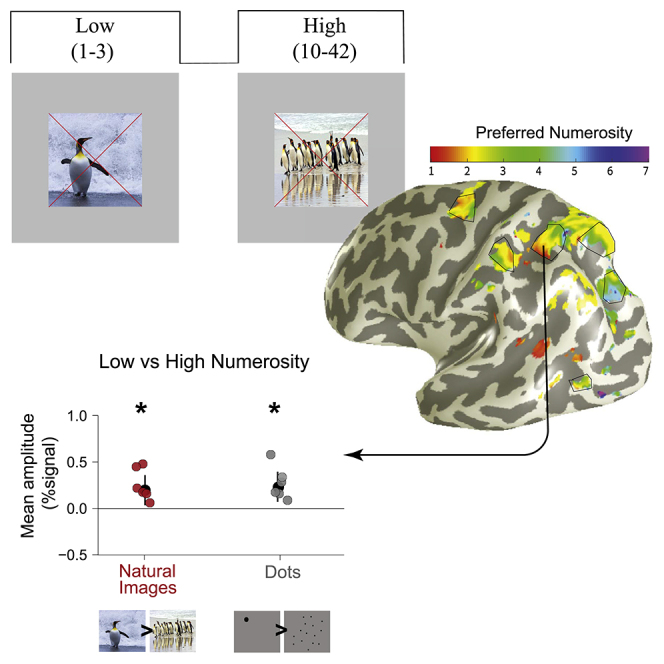

## Introduction

Animals and humans are able to quickly, and effortlessly estimate the number of items in a set: their numerosity. Identifying the numerosity of objects in the environment is essential for numerous behaviors such as foraging, mating, and navigation and are therefore considered to be of adaptive value. In humans, the basic ability to perceive non-symbolic quantities is linked with the development of mathematical cognition, and are therefore suggested to hold educational significance (Anobile et al., 2016; Halberda et al., 2008; Malone et al., 2019).

Studying numerosity perception is complex since different sets cannot be different in numerosity alone. Increase in numerosity goes hand in hand with increase in some dimension of sensory input. For example, as the average number of items increase, so is the total area they occupy. Therefore, most numerosity studies use multiple well-controlled artificial stimuli, most commonly dots, trying to account for effects of other continuous magnitudes (Burr and Ross, 2008; Clayton et al., 2015; Dakin et al., 2011; Franconeri et al., 2009; Gebuis et al., 2016; Gebuis and Reynvoet, 2012a, 2012b; Harvey and Dumoulin, 2017a).

Using well-controlled artificial stimuli, electrophysiology studies found neurons that are specialized for numerosity in both animals and humans (Ditz and Nieder, 2015; Kutter et al., 2018; Nieder et al., 2002; Tsouli et al., 2022). These neurons show tuned response curves: they exhibit a pick in response for a specific numerosity, and this response decreases as the numerical distance grows. Human neuroimaging showed that the tuned neural populations are organized in a network of topographic maps (Cai et al., 2021; Harvey et al., 2013; Harvey and Dumoulin, 2017b; Hofstetter et al., 2021). The tuning features of the numerosity neurons follow important aspects of our numerosity perception and behavior, such as the numerical distance and size effects (Cai et al., 2021; Nieder and Dehaene, 2009; Tsouli et al., 2022), and in monkeys, their activity was linked to numerical skills (Nieder et al., 2002; Nieder and Miller, 2004).

Even though the use of artificial stimuli made important contributions of our understanding of numerosity perception, artificial stimuli have little ecological validity. Several studies suggest a limited ability to extrapolate to real-life situations from artificial stimuli and tasks (Carandini et al., 2005; Kayser et al., 2004). For example, performance of visual tasks in the absence of attention differs substantially with artificial versus natural stimuli (Li et al., 2002), and single neuron descriptions derived from artificial stimuli do not always extrapolate well to natural stimuli (David et al., 2004). Therefore, several investigators have called for more natural experimental protocols using natural images and tasks (Felsen and Dan, 2005; Kayser et al., 2004; Olshausen and Field, 2005; Yuille and Kersten, 2006).

Here, we ask whether numerosity-tuned neural responses also respond to real-world natural images. To that end, we utilize the recently uncovered network of visual topographic numerosity maps, and evaluate whether numerosity-selective responses can be driven by viewing natural images, i.e., 2D photographs of real-world scenes showing a variety of real-world items with different numerosities. Our results provide evidence indicating that the numerosity-tuned neural populations (Cai et al., 2021; Harvey et al., 2013; Harvey and Dumoulin, 2017b; Tsouli et al., 2022) show similar properties of selectivity when viewing, briefly, an image from a real-world visual scene.

## Results

**Figure 1:**
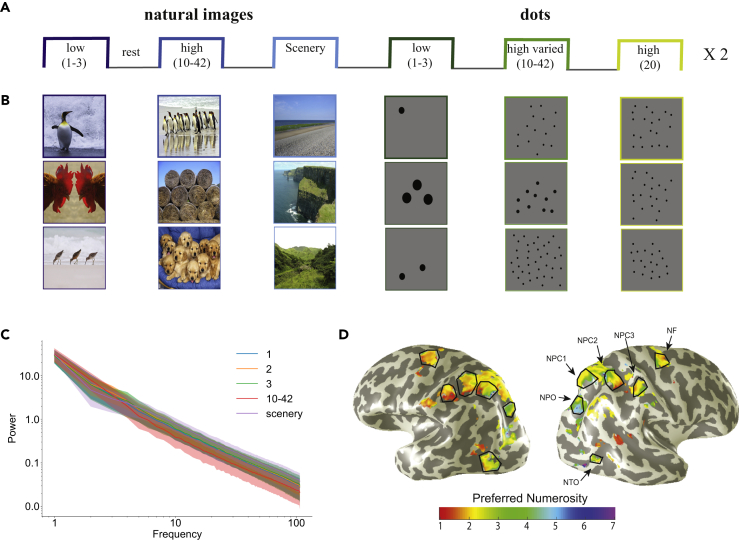
Study design (A) A schematic representation of the block design, consisting of 6 visual categories of either natural images or dots. The blocks were presented in a random order, and each category was repeated twice in one functional run. Each block lasted 15 s followed by a 15 s presentation of mean-luminance. (B) Examples of the stimuli. Stimuli included presentation of natural image or dots, divided into different categories based on their numerosities. In the natural images categories, the images were randomly picked out of a pre-selected pool. (C) Though natural images vary along many dimensions, the power spectrum of the natural images containing either 1, 2, or 3 main objects, 10–42 main objects and scenery are similar and following a similar amplitude spectra across categories (Field, 1987). The shaded errorbars show the SD across images of an image category. (D) Example of the numerosity maps of one participant. Within these maps, we localized the neural populations tuned to numerosity 1 to 3.

Seven participants were scanned at ultra-high field (7T) MRI while watching a series of natural images or artificial dots organized in blocks. The stimuli were comprised of 6 categories (Figures 1A and 1B): (1) natural images with one to three main objects, (2) natural images with high numerosity (mean number of objects was 19.42 with a SD of 8.8), (3) natural images of scenery, (4) computerized stimuli consisting of one to three dots, (5) 20 dots (constant high numerosity), (6) 10–42 dots with a similar frequency of the numerosity of condition (2) (high numerosity but not constant). The natural images varied across many dimensions (color, texture, etc.), but are similar in their power spectra, i.e. all natural images showed the expected 1/f distribution with natural images (Field, 1987) (Figure 1C). Participants were asked to keep fixation and respond when the same image or dot display was presented repeatedly (1-N back test). No numerosity judgment was required. In a separate experiment, we localized the participants’ numerosity maps (Figure 1D).

**Figure 2:**
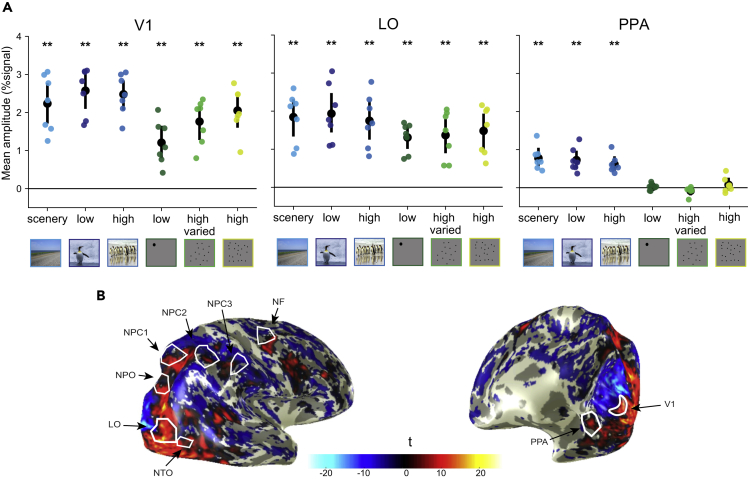
Neural responses to natural images in selective visual areas (A) Averaged neural response in three control regions of the visual system: primary visual cortex (V1), lateral occipital area (LO), and parahippocampal place area (PPA). As expected, in the visual regions the natural images in all of the three categories produced high responses. The dots stimuli produced high responses in V1 and LO (lateral occipital area) but not in the PPA (parahippocampal place area). Colored dots represent the mean response of each participant. Black circles represent median of the data. Error bars show the SD of observations. ∗∗ indicates p < 0.01 following one-sided Wilcoxon signed rank test and FDR correction for multiple comparisons. (B) An example of cortical responses to natural images of scenery in one participant. The statistical map shows the t-values of a contrast between the scenery blocks and rest. The borders of the numerosity maps and the visual control ROIs are shown in white. The responses in the numerosity maps are mixed and includes negative or non-significant responses to viewing of the natural images (i.e., irrespective of numerosity).

The current study aimed to take advantage of the high ecological validity of natural images. However, the use of natural images limits our ability to control for specific visual aspects (such as color, contrast, density, etc.). Therefore, our first step was to check the neural responses to our selected stimuli and study design using three visual regions that have known selectivity characteristics: the primary visual area (V1); the lateral occipital area (LO), specialized in objects shape recognition (Kourtzi and Kanwisher, 2001; Malach et al., 1995); and the parahippocampal place area (PPA), specialized in scenery recognition (Epstein and Kanwisher, 1998). As expected, in V1 and LO, we found an overall high response across all the dots and natural images categories. In the PPA, only the natural images showed a significant response, which is expected since all image categories contain scenery information (Figure 2A; one-sided Wilcoxon signed rank test, p < 0.05, FDR corrected).

Next, we identified the network of numerosity maps in all of our participants (Figure 1D) and selected the neural populations with tuned responses to numerosity 1 to 3. The use of natural images inherently results in a mix of visual neurons responding to diverse visual dimensions. For example, within the partial lobe, object size selective neural populations were found to be intertwined with the numerosity-tuned neural populations (Harvey et al., 2015; Tsouli et al., 2022). Furthermore, the network of numerosity maps overlaps to some degree with visual field maps (Wandell et al., 2007) and the relation between the two is unclear (Harvey et al., 2013). Therefore, also properties of experimental stimuli design, such as stimulus size, in combination with known neural properties, such as visual field maps and center-surround, might affect the overall measured neural response in some parts of the numerosity maps (Wandell et al., 2007). In other words, natural images elicit a broad response that includes many other neurons beyond those tuned to numerosity.

**Figure 3:**
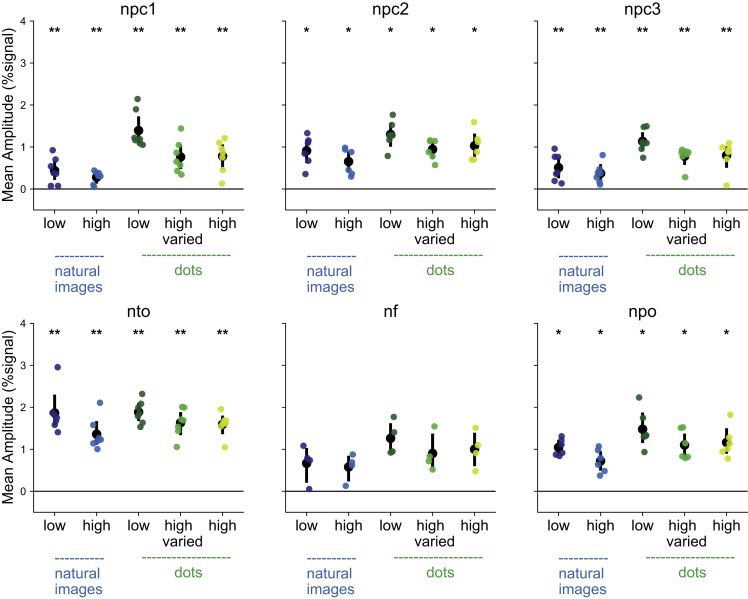
Neural responses to natural images and dots in the numerosity maps The natural scenery category was used to remove cortical points where the main driven response might be due to the design of the presented stimuli. Excluding these cortical points, significant positive responses to all natural images categories were found across all the numerosity maps, except for the frontal maps (NF). Colored dots represent the mean response of each participant. Black circles represent median of the data. Error bars show the SD of observations. ∗ indicates p < 0.05, ∗∗p < 0.01 following one-sided Wilcoxon signed rank test and FDR correction for multiple comparisons.

In order to minimize the influence of neural responses that is mostly driven by other stimulus features (such as expected negative responses at the borders of the presented images), we excluded from further analyses cortical points where the responses to the natural images of scenery, where no clear numerosity is present, were negative or non-significant (t < 1.96 in a GLM analysis contrasting scenery images with rest; Figure 2B). Taking into consideration, cortical points that show responses to the natural images of scenery increased the averaged neural responses in the other two categories of the natural images (low and high numerosity). In all of the numerosity maps but the frontal maps, the neural responses were significantly positive (Figure 3). However, even without a threshold based on the scenery, we observed similar neural responses though with decreased significance (Figure S1).

**Figure 4:**
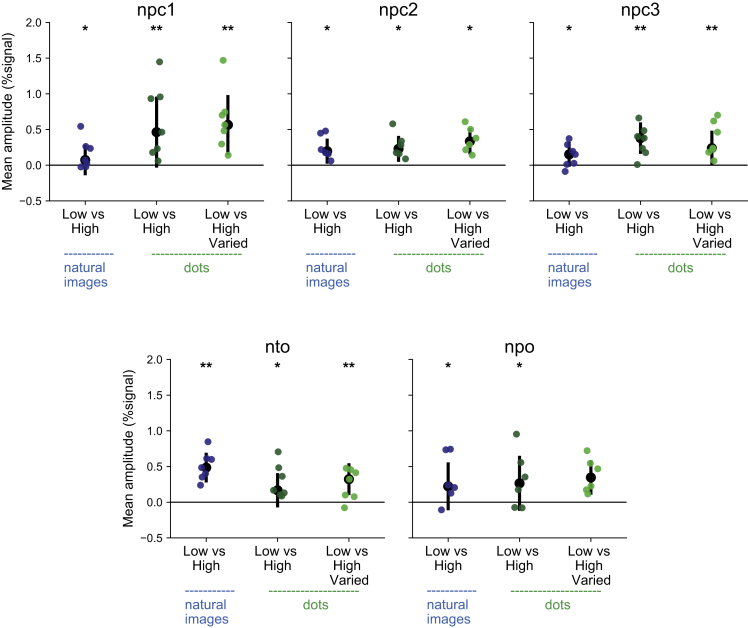
Preferred neural responses to low numerosity presented in natural images The averaged neural responses to low numerosity were significantly higher than the averaged responses to high numerosity, indicating the neural populations were influenced by the numerosity presented in the natural images and suggest that the response follows a tuned response curve. Colored dots represent the mean response of each participant. Black points represent the median of the data. Error bars show the SD of the observations. ∗ indicates p < 0.05, ∗∗p < 0.01 following one-sided Wilcoxon signed rank test and FDR correction for multiple comparisons.

Last, we asked whether the neural populations within the numerosity maps show their known tuning characteristic when presented with different number of objects in natural images, i.e., preferred lower numerosities over higher numerosities. To that end, we compared the responses in the low numerosity blocks to the responses in the high numerosity blocks. We note that this analysis does not reveal the entire tuning function, but is indicative and consistent with tuning. Due to the low responses in both dots and natural images categories, the frontal maps were excluded from this analysis. Significantly higher responses to low vs high numerosity were found in all of the maps (one-sided Wilcoxon signed rank test, p < 0.05, FDR corrected; Figure 4). These results indicate that in accordance with the selected neural population’s preferred numerosity, as was found using an artificial stimulus, a different response is gained based on the numerosity of objects contained in the natural images.

## Discussion

An important stage in identifying the biological mechanism of numerosity perception was the findings of neurons that are tuned to numerosity (Ditz and Nieder, 2015; Nieder et al., 2002; Nieder and Miller, 2004; Piazza et al., 2004). The tuned response properties of numerosity-selective neurons follow weber’s law, and are thought to underlie important aspects of numerosity perception such as the distance effect and the size effect (Nieder, 2016; Tsouli et al., 2022). Tuned numerosity responses were also found to emerge in trained and untrained neural networks (Kim et al., 2021; Nasr et al., 2019). In the study by Nasr et al., neural networks were trained to classify objects from natural images. Following training, the networks were able to correctly identify the numerosity of dots displays, suggesting that numerosity-tuned responses are based on mechanisms inherent to the visual system (Nasr et al., 2019). Though numerosity neural selectivity greatly advanced our understanding of numerosity perception, their responses to real-world images were not tested before. The results of our study expand the ecological validity of numerosity-selective neurons and therefore reinforce their role in numerosity perception in daily life.

How numerosity is extracted from the visual scene is still unknown. Some proposals suggest that numerosity may be extracted from different spatial frequency representations of early visual cortex, either ratio of high and low spatial frequencies (Dakin et al., 2011) or aggregate power (Paul et al., 2022). Given that the power spectra are similar for the different images used in this study, these proposals would not work on the raw natural images per se, but may still be feasible in combination with object segmentation (Dehaene and Changeux, 1993; Nasr et al., 2019) and/or more basic second-order filters (Johnson and Baker, 2004).

In real-world environments, the numerosity being identified may depend on which objects, or their features, are at the focus of attention. For example, one can quickly estimate three bagels and disregard the one platter that carries them. The numerosity of an image of a four-leg chair can be interpreted as one, but focusing on the legs of the chair may result in numerosity perception of four. Using simple stimuli of black and white dots, we have recently found that attention, to some feature of a presented stimuli (e.g., shape), is needed in order to drive numerosity responses (Cai et al., 2022). The numerosity-tuned neural populations were responsive to the numerosity of the objects being attended, and not to the overall quantity of objects being presented. In the current study, participants were fixating at the center of natural images and were engaged in a memory task which also did not require any numerosity estimation but did focus the participants attention toward the natural image. Within the short presentation time of each image, the main objects of the natural can already be recognized (Thorpe et al., 1996). The finding of different response magnitude between low and high numerosity in the natural images categories (Figure 4) suggests that the numerosity identified by our participants was of the number of main objects in the image, while ignoring either background or other sub-features of the objects in the images (e.g., the number of legs in an image of 3 dogs). In the block design, the task was the same for natural images and dots (one back task). However, during the localizer scans of the numerosity maps, the participants performed a different task (color judgment). However, in all conditions, the participants paid attention toward the stimuli and did not perform a numerosity judgment. Therefore, we do not believe that a difference in task between the localizer and experiment explains our results.

### Limitations of the study

The study of natural images holds an inherent limitation of uncontrolled visual properties such as contrast, color, etc. Therefore, the measured neural response is the outcome of many types of neurons preferring different dimensions of the visual scene. We have tried to limit the influence of neural responses related to stimuli design by thresholding the cortical points based on their responses to natural images of scenery (i.e., irrespective of numerosity). This, of course, cannot rule out other types of stimuli-driven responses. However, by selecting cortical points with known numerosity-tuned neural populations (Cai et al., 2021; Harvey et al., 2013; Harvey and Dumoulin, 2017b), we were able to overcome some of this limitation and find neural responses to natural images that differentiated in the numerosity of items they presented. That said, the results are similar without the thresholding based on natural images of scenery though with decreased significance (Figure S2).

In addition, we did not reveal the entire tuning curves elicited by viewing natural images, but only the comparison between preferred numerosities and baseline. This is due to our simple but robust design. We did not opt for a full pRF design due to concerns of SNR, stimulus confounds, and availability of enough natural images especially for the higher numerosities in existing datasets. However, we feel revealing the entire tuning curve is not necessary to prove that neural populations within the numerosity maps respond to the numerosity in natural images.

Furthermore, we used images of natural scenes, i.e. photographs of real-world scenes displayed on a computer screen. Though they are more natural and ecological valid than images of dots, they are not the same as the three-dimensional world we encounter and interact with on a daily base. Therefore, this study is a step in the direction of more ecological valid stimuli, but not identical to daily life experiences.

In addition, we were unable to find significant responses to all of the dots’ categories and to the categories of the natural images in the numerosity maps of the frontal lobe (Figure S1). We believe that this result is due to lack of statistical power since only four participants in our cohort showed topographic numerosity maps in their frontal lobes. The lack of significant responses to the presentation of numerous dots suggests that the low responses in the frontal maps are not due to the types of stimuli (i.e., dots vs. natural images), but are the result of low statistical power. Future studies that wish to focus on the neural responses in these maps should take into consideration the intrinsic variability in the presentation of the topographic numerosity maps between participants and across the cortex.

## STAR★Methods

### Key resources table

**Table:** 

REAGENT or RESOURCE	SOURCE	IDENTIFIER
**Software and algorithms**		

Vistasoft	(http://vistalab.stanford.edu/software/),	NA

### Resource availability

#### Lead contact

Further information and requests for resources should be directed to and will be fulfilled by the lead contact, Prof. Serge Dumoulin (s.dumoulin@spinozacentre.nl).

#### Material availability

This study did not generate new unique reagents.

### Experimental model and subject details

#### Participants

Seven participants participated in the study (3 females, 2 left handed, mean age 34, age range 25–48). All participants had normal or corrected-to-normal visual acuity. All experimental procedures were approved by the ethics committee of VU Amsterdam. Participants gave informed consent.

### Method details

#### Stimuli and experimental design

Visual stimuli were presented on a 69.84 × 39.29 cm LCD screen (Cambridge Research Systems) behind the MRI bore. Stimuli were viewed through a mirror attached to the head coil. The total distance from the attached mirror to the display screen was 220 cm. The display resolution was 1920 × 1080 pixels. A button box recorded behavioral responses. Visual stimuli were generated or viewed in Matlab using PsychToolbox (Kleiner et al., 2007). A large diagonal cross composed of thin red lines was displayed consistently across the entire screen, serving as a fixation marker.

The experiment consisted of 6 categories of stimuli (Figure 1A): (1) natural images with one to three main objects; (2) natural images with high numerosity (mean number of objects was 19.42 with a standard deviation of 8.8; (3) natural images of scenery; (4) computerized stimuli consisting of one to three dots; (5) 20 dots; (6) 10–42 dots with at a similar frequency to the numerosity of (2). The images vary across many dimensions (colour, texture, etc.) but are similar in powerspectra (expected 1/f distribution as known with natural images)(Field, 1987) (Figure 1B).

The natural images were selected from the ImageNet Large Scale Visual Recognition Challenge (ILSVRC) (https://www.kaggle.com/c/imagenet-object-localization-challenge/data?select=LOC_synset_mapping.txt). The images were resampled to fit the central 2° (diameter) of the visual field.

The stimuli in each category were presented in blocks of 15 seconds, followed by 15 seconds of gray background (rest periods). In each block, 30 natural images were randomly chosen out of their pre-selected pool (the pool included 93 images of 1 main object, 95 images of 2 objects ,70 images of 3 objects, 70 images of the high numerosity and 61 images that showed scenery views). Each natural image or dots display were presented for 300 ms, with 200 ms of gray background between displays. The 6 categories were presented twice in each trial. Their order was randomly assigned.

The dots were randomly generated where either constant area of dots or the dot size was kept constant. Similar to previous numerosity experiments (Cai et al., 2021; Harvey et al., 2013), in each presentation the dots were placed in a new, random position, and the individual items were distributed roughly homogeneously across the stimulus area.

Participants were asked to keep fixation and respond when the same image or dot display was presented repeatedly (1-N back test). No numerosity judgment was required.

#### MRI acquisition

MRI data was acquired on a 7T Philips Achieva scanner. Functional runs were acquired using a 32 channel head coil with the following parameters: isotropic resolution of 1.7 mm^3^; TR/TE = 1500/22.5; flip angle = 65; multiband factor = 3. Data included 57 slices, 248 TRs that lasted 6:24 min. The natural images experiment included 7 functional trials (runs) that were acquired in one scanning session.

We used T1 weighted images that were acquired prior to our experiment. These scans were acquired with MP2RAGE sequence with the following parameters: TR = 6.8 ms; TE = 2.3 ms, flip angle = 5°; isotropic resolution of 0.8^3^, SENSE factor = 2; slices = 205.

#### Pre-processing

Data was analysed using *fMRIPrep* 20.1.1 (Esteban et al., 2019), which is based on *Nipype* 1.5.0 (Gorgolewski et al., 2011, 2017). The T1-weighted (T1w) image of each participant was corrected for intensity non-uniformity (INU) with N4BiasFieldCorrection (Tustison et al., 2010), distributed with ANTs 2.2.0 (Avants et al., 2008), and used as T1w-reference throughout the workflow. The T1w-reference was then skull-stripped with a *Nipype* implementation of the antsBrainExtraction.sh workflow (from ANTs), using OASIS30ANTs as target template. Brain tissue segmentation of cerebrospinal fluid (CSF), white-matter (WM) and gray-matter (GM) was performed on the brain-extracted T1w using fast (FSL 5.0.9, RRID:SCR_002823, (Zhang et al., 2001)).

For each of the BOLD runs acquired per subject (across all conditions and sessions), the following preprocessing was performed: First, a reference volume and its skull-stripped version were generated using a custom methodology of fMRIPrep. A deformation field to correct for susceptibility distortions was estimated based on fMRIPrep’s fieldmap-less approach. The deformation field is that resulting from co-registering the BOLD reference to the same-subject T1w-reference with its intensity inverted (Huntenburg, 2014; Wang et al., 2017). Registration is performed with antsRegistration (ANTs 2.2.0), and the process regularized by constraining deformation to be nonzero only along the phase-encoding direction, and modulated with an average fieldmap template (Treiber et al., 2016). Based on the estimated susceptibility distortion, a corrected EPI (echo-planar imaging) reference was calculated for a more accurate co-registration with the anatomical reference. The BOLD reference was then co-registered to the T1w reference using bbregister (FreeSurfer) which implements boundary-based registration (Greve and Fischl, 2009). Co-registration was configured with six degrees of freedom. Head-motion parameters with respect to the BOLD reference (transformation matrices, and six corresponding rotation and translation parameters) are estimated before any spatiotemporal filtering using mcflirt (FSL 5.0.9, (Jenkinson et al., 2002)). The BOLD time-series were resampled onto the following surfaces (FreeSurfer reconstruction nomenclature): fsnative, fsaverage. The BOLD time-series were resampled onto their original, native space by applying a single, composite transform to correct for head-motion and susceptibility distortions.

The rest of the analysis was done using MATLAB and vistasoft (http://vistalab.stanford.edu/software/). For each subject, the first 8 timeframes were discarded from the functional scans. In order to increase signal strength, data from all recording points (voxels) across cortical thickness were collapsed and averaged onto the nearest point on the cortical surface (Harvey et al., 2013; Hofstetter et al., 2021).

#### Localizing numerosity maps

We used data from former numerosity experiments to identify the numerosity maps of our participants (Figure 1D). The data was acquired for other studies and included a similar design to that previously described (Harvey et al., 2013; Harvey and Dumoulin, 2017a, 2017b; Tsouli et al., 2022; Cai et al., 2021; Hofstetter et al., 2021). In short, a sequence of numerosity stimuli consisting of 1 to 7 dots were first presented in ascending order, followed by a long period with a baseline presentation of 20 dots, then followed by the same sequence in descending order and another identical baseline period. This sequence was repeated four times for each fMRI scan run.

Numerosity stimuli consisted of a group of dots, presented in the central 4° (diameter) of the visual field, with a constant total surface area. Dots were randomly positioned at each presentation within this area. Therefore, contrast energy was equally distributed across the stimulus area for all numerosities. Each numerosity presentation that contained the same number of dots was placed in a new, random position, so no specific visual position was associated with any numerosity. To prevent perceptual grouping, individual items were distributed roughly homogeneously across the stimulus area. Dot patterns were presented briefly (300 ms) to ensure participants did not have time to count. A new random pattern was presented every 650 ms, with 350 ms presentation of a uniform gray background between dot pattern presentations. This was repeated six times, over 3900 ms, corresponding to two fMRI volume acquisitions (TR), before the numerosity changed. The numerosity stimuli were displayed as black dots, where in 10% of numerosity presentations the dots were shown in white. Participants were asked to fixate at the red cross in the center and press a button when whites dots were shown to ensure they were paying attention to the stimulus during fMRI acquisition. No numerosity judgements were required.

Tuned numerosity responses were estimated using pRF modeling (Dumoulin and Wandell, 2008; Harvey et al., 2013; Harvey and Dumoulin, 2017b). The pRF model describes the averaged tuning of the underlying neural populations using logarithmic Gaussian functions. These Gaussian functions are characterized by two parameters: preferred numerosity (mean of the Gaussian) and tuning width (standard deviation of the Gaussian).

#### Control regions of interests (ROIs)

Three regions of the visual cortex (V1, parahippocampal place area (PPA), and lateral-occipital object area (LO)) were selected as controls. Bilateral PPA and LO were defined using brain atlases (Wang et al., 2015; Weiner et al., 2018). Bilateral V1 were defined using retinotopy maps of each participant that were previously acquired for other studies (Harvey and Dumoulin, 2011; Oliveira et al., 2022). The retinotopy maps were analysed using the pRF method (Dumoulin and Wandell, 2008). V1 were manually defined and restricted to 2° of visual angle.

### Quantification and statistical analysis

Using vistasoft (http://vistalab.stanford.edu/software/), a general linear model was used to test the neural responses to natural images of scenery along the cortex. Moreover, we averaged the neural responses of all categories per regions of interest (control ROIs and numerosity maps) and participants. Within the numerosity maps we pre-selected the neural populations that showed preferred response to numerosity 1–3. For each type of numerosity map (i.e., npc1, npc2 etc.) we averaged the responses across both hemispheres. One-sided Wilcoxon test, followed by FDR correction for multiple comparisons was used to test for a significant positive BOLD response. These statistical tests were computed using MATALB.

## Data Availability

• Data reported in this paper will be shared by the lead contact upon request. • This paper does not report original code. • Any additional information required to reanalyze the data reported in this paper is available from the lead contact upon request.
