# Supplementary material for: Assessing the ecological validity of numerosity-selective neuronal populations with real-world natural scenes
Source: iScience. 2022 Oct 4;25(10):105267. doi: 10.1016/j.isci.2022.105267 (PMC9579010; doi:10.1016/j.isci.2022.105267)
Supplement: Document S1. Figures S1 and S2 [file mmc1.pdf]

iScience, Volume *25*

Supplemental information

Assessing the ecological validity of numerosity-selective neuronal populations with real-world natural scenes

Shir Hofstetter and Serge O. Dumoulin

Supplemental Information:

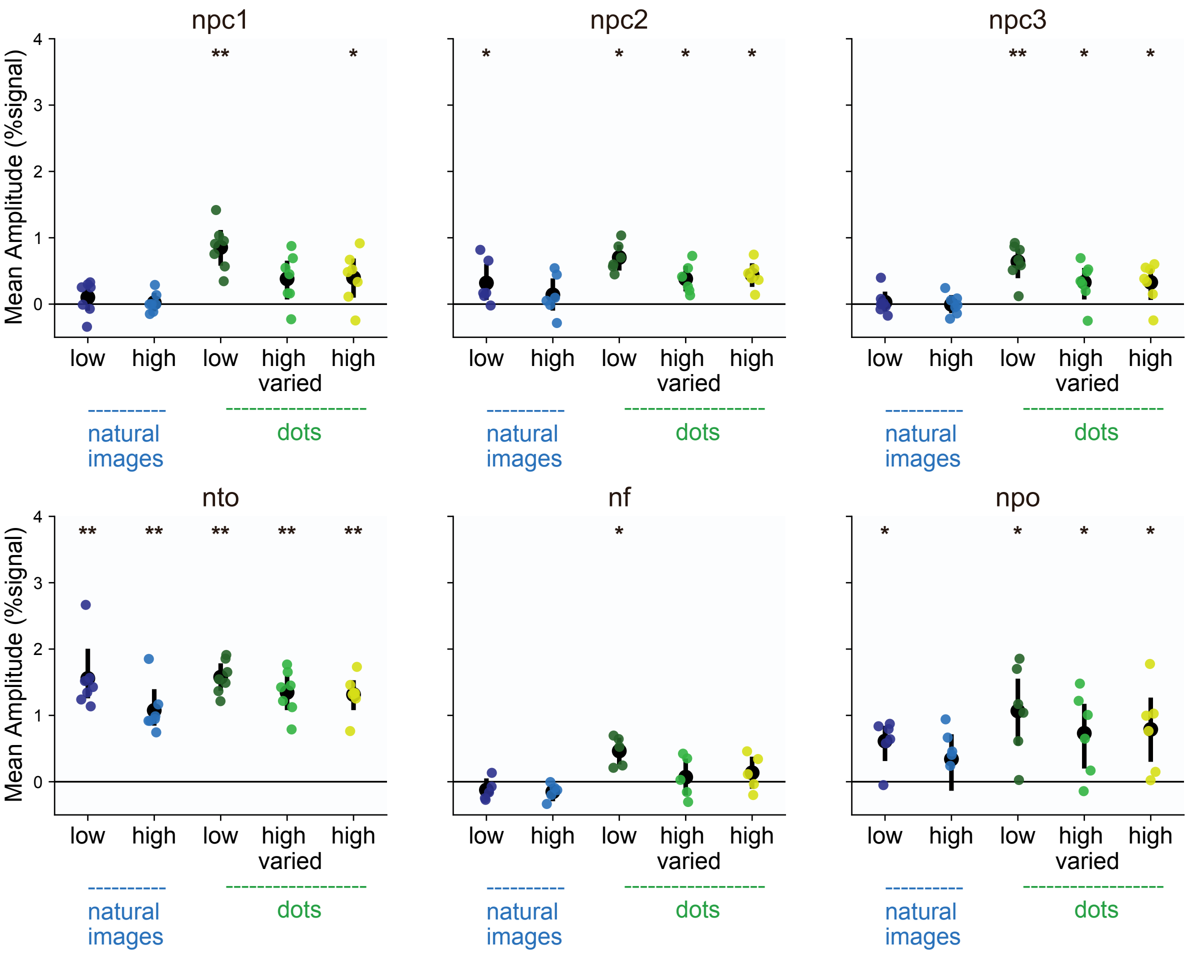

Figure s1 (Related to Figure 3): Neural responses to natural images and dots representations in numerosity populations selective to low numerosity (1-3). The neural responses in each numerosity map were averaged across the cortical points with numerosity preference of 1 to 3. Significant positive responses to all dots categories were found across all the numerosity maps, except for the frontal maps (NF). Significant responses to some of the conditions of the natural images were found in NTO, NPO and NPC2 maps. Colored dots represent the mean response of each participant. Black circles represent median of the data. Error bars show the standard deviation of observations. * indicates $p<0.05$, ** $p<0.01$ following one-sided Wilcoxon signed rank test and FDR correction for multiple comparisons.

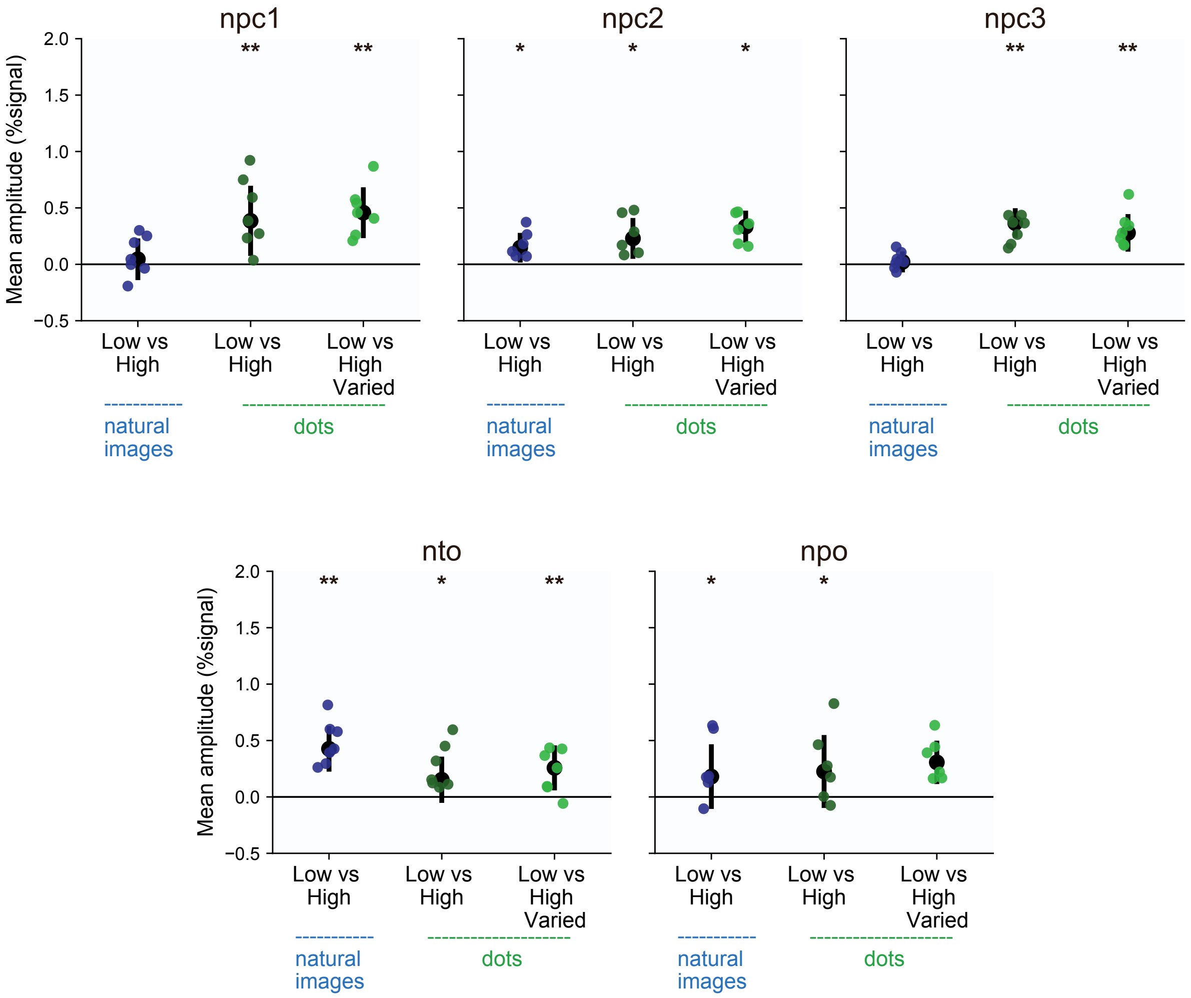

Figure s2 (Related to Figure 4): Preferred neural responses to low numerosity presented in natural images. The neural responses in each numerosity map were averaged across cortical points with numerosity preference of 1 to 3 (without a second restriction based on the neural responses to scenery). In three of the numerosity maps the averaged neural responses to low numerosity were significantly higher than the averaged responses to high numerosity. Colored dots represent the mean response of each participant. Black points represent the median of the data. Error bars show the standard deviation of the observations. * indicates $p<0.05$, ** $p<0.01$ following one-sided Wilcoxon signed rank test and FDR correction for multiple comparisons.
